# Enhancing gesture recognition with multiscale feature extraction and spatial attention

**DOI:** 10.1371/journal.pone.0324050

**Published:** 2025-06-09

**Authors:** Jingpeng Lei

**Affiliations:** Department of Information Technology, Anhui Vocational College of Defense Technology, Lu’an, Anhui Province, China; New York University Abu Dhabi, UNITED ARAB EMIRATES

## Abstract

Gesture recognition technology is a pivotal element in human-computer interaction, enabling users to communicate with machines in a natural and intuitive manner. This paper introduces a novel approach to gesture recognition that enhances accuracy and robustness by integrating multiscale feature extraction and spatial attention mechanisms. Specifically, we have developed a multiscale feature extraction module inspired by the Inception architecture, which captures comprehensive features across various scales, providing a more holistic feature representation. Additionally, We incorporate a spatial attention mechanism that focuses on image regions most relevant to the current gesture, thereby improving the discriminative power of the features. Extensive experiments conducted on multiple benchmark datasets demonstrate that our method significantly outperforms existing gesture recognition techniques in terms of accuracy.

## Introduction

Gesture recognition, as a key technology in the field of human-computer interaction, has long been a hot topic in computer vision and machine learning research [1]. This technology facilitates communication with computers through the recognition and interpretation of human gestures, making interaction more intuitive and natural. With the proliferation of smart devices and enhanced computational capabilities, gesture recognition has been widely applied in areas such as game control, smart home operations, and assisted driving systems, significantly enriching user interaction experiences [2].

Despite its advancements, the development of gesture recognition technology still faces several challenges. Firstly, the high diversity of gestures and individual differences demand that recognition systems possess exceptional flexibility and adaptability [3]. Additionally, in practical applications, complex backgrounds and varying lighting conditions often negatively impact recognition accuracy. These factors contribute to a significant gap between the performance of gesture recognition in real-world settings and laboratory conditions [4].

In response, this paper proposes a new gesture recognition method that integrates multi-scale feature extraction with spatial attention mechanisms [5]. Multi-scale feature extraction captures gesture details and global information at different levels, while the spatial attention mechanism further enhances the model’s focus on key parts of gestures, significantly improving recognition performance in complex backgrounds. While multi-scale feature extraction and attention mechanisms are well-known in general computer vision, their application to 3D hand gesture recognition remains limited. This method has been validated on multiple standard datasets, and experimental results show that it increases accuracy across diverse gesture recognition tasks. The multi-scale feature extraction and attention mechanisms contribute to the model’s adaptability and potential robustness, suggesting promising applications in real-world gesture recognition systems. These advancements open new possibilities for the practical application and further development of gesture recognition technology.

This research primarily aims to address challenges related to gesture diversity and complex backgrounds in recognition systems. The proposed method aims to enhance recognition accuracy and robustness by integrating multi-scale feature extraction and spatial attention mechanisms. This approach enables a more flexible and adaptive system capable of handling diverse gestures and varying environmental conditions.

The contributions of this study are summarized as follows:

1. **Applied multi-scale feature extraction to hand gesture recognition**: This module has been specifically adapted to capture both local details and global structures of hand gestures by processing image information at multiple scales. Its adaptability improves the model’s ability to handle diverse gesture shapes and complex backgrounds, demonstrating its effectiveness in 3D hand gesture recognition tasks.
2. **Implemented an adaptive spatial attention mechanism for hand gesture recognition**: This mechanism is designed to dynamically adjust focus on critical gesture features, enhancing the model’s discriminative capability and robustness. It improves recognition accuracy while maintaining computational efficiency, highlighting its applicability in real-world gesture recognition scenarios.

This paper addresses the challenge of gesture recognition by proposing a novel approach that leverages multiscale feature extraction and spatial attention mechanisms. The rest of the paper is organized as follows: In the Related Work section, we review related work in the field of gesture recognition, discussing various methods and their limitations. The Methodology section presents the methodology, detailing the proposed approach and its key components. In the Experiments section, we describe the experimental setup, datasets, and evaluation methods used to assess the effectiveness of the proposed method, present an analysis of the attention module’s impact on performance, and compare the proposed method with state-of-the-art techniques. Finally, the Conclusion section concludes the paper, summarizing the contributions and suggesting potential directions for future research.

## Related work

Gesture recognition has seen significant advancements in recent years, particularly with the integration of deep learning technologies. This section reviews the key developments and highlights the theoretical and technological foundations for our study, addressing the limitations of existing methods and emphasizing the advantages of our approach.

### Applications of deep learning in gesture recognition

Deep learning has significantly transformed gesture recognition, with architectures like Convolutional Neural Networks (CNNs) and Recurrent Neural Networks (RNNs) enhancing different aspects of performance. CNNs, as demonstrated in works by [6] and [7], are particularly effective for their spatial feature extraction capabilities, making them suitable for analyzing static images and adapting to video data by treating frames as stacked images. However, they often require extensive labeled datasets and struggle with temporal dynamics of gestures [8, 9]. On the other hand, RNNs excel at processing temporal data sequences, capturing gesture dynamics over time. However, they can suffer from issues like vanishing or exploding gradients, which complicate training and limit their efficacy in longer sequences [10, 11].

The incorporation of attention mechanisms has further improved gesture recognition performance by enabling models to focus on the most relevant parts of the input data. Models like Transformer-based architectures have demonstrated superior accuracy in complex gesture tasks by utilizing both spatial and temporal attention [12, 13]. In gesture recognition, attention mechanisms not only enhance spatial feature extraction but also help capture subtle temporal variations in gestures [14, 15]. Similarly, adaptive attention networks have been proposed to focus on relevant features dynamically, improving recognition accuracy in diverse settings [16]. Our approach leverages multiscale feature extraction within a CNN framework combined with spatial attention mechanisms, enhancing the model’s capability to focus on both global and local gesture features, thus improving efficiency and accuracy [17, 18].

### Multiscale feature extraction in gesture recognition

Capturing the full range of gesture dynamics and details is challenging with single-scale feature extraction due to the diversity of spatial and temporal characteristics present in gestures. Multiscale feature extraction techniques, such as those implemented in the Inception network, effectively address this challenge by using convolutional kernels of different sizes concurrently within the same layer, enabling simultaneous extraction of features across various scales [19]. For instance, Guo *et al*. [20] and Chen *et al*. [21] demonstrated that multiscale feature extraction can significantly improve hand pose estimation, which is closely related to gesture recognition.

Recent methods have introduced advanced multiscale networks that not only consider different spatial scales but also integrate temporal scales, allowing models to adapt more effectively to dynamic gestures [22, 23]. Techniques such as dual attention mechanisms have also been applied to multiscale feature extraction, where the model focuses on both spatial and temporal features concurrently. In addition, efficient multiscale extraction has been achieved through the use of lightweight architectures that maintain high accuracy while reducing computational load, making real-time deployment more feasible [24, 25].

Despite their success, traditional multiscale methods often lead to increased computational demands and model complexity, which can hinder real-time performance. To address these challenges, our approach optimizes multiscale feature extraction by balancing computational demands with the need for detailed and precise feature representation. This optimization maintains high accuracy while enhancing efficiency, making it well-suited for real-time operational environments [26, 27]. By combining multiscale techniques with adaptive attention mechanisms, our model achieves robust gesture recognition across diverse settings, effectively addressing the limitations of previous methods and providing significant advancements in both static and dynamic environments [17, 18].

### Application of attention mechanisms in gesture recognition

Attention mechanisms have emerged as essential tools for enhancing neural network performance, particularly in gesture recognition, where they improve model accuracy by focusing on crucial image areas. This enhancement of the model’s ability to learn and prioritize essential gesture features, as demonstrated by [14], helps significantly in handling the complex dynamics of gestures. Despite their advantages in feature discernment, these mechanisms heavily depend on the architecture of the underlying feature extraction system. Improper calibration can lead to misalignment between the focus areas and the most informative parts of a gesture, causing the model to overlook subtle yet crucial gesture dynamics. To address this, our method incorporates a dynamically adjusting spatial attention mechanism that aligns focus with the contextual relevance of gesture features, ensuring optimal resource allocation and improved recognition accuracy. This dynamic adjustment not only mitigates the limitations found in static attention systems but also enhances the ability of our model to handle complex, real-time gesture recognition scenarios effectively.

## Methodology

### Model overview

**Fig 1:**
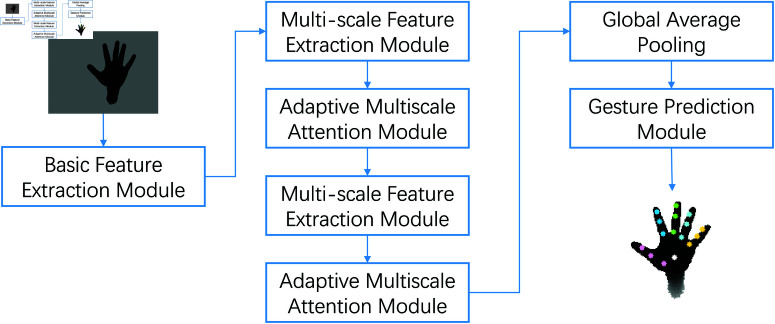
Overview of the proposed gesture recognition model architecture.

Our proposed gesture recognition model comprises three primary modules: the Basic Feature Extraction Module, the Multi-Scale Feature Extraction Module, and the Adaptive Multi-Scale Attention Module. These modules work collaboratively to achieve efficient gesture recognition. The main framework is illustrated in Fig 1.

- **Basic feature extraction module**: This module extracts fundamental visual information from the input 128×128 pixel depth map using multiple layers of convolutional networks. It captures low-level features such as edges and corners, laying the groundwork for more complex feature analysis.
- **Multi-scale feature extraction module**: By applying convolutional kernels of various sizes at different levels, this module captures details at multiple scales. This multi-scale processing allows the model to understand both fine local details and the global structure of gestures across a broader area, enhancing its adaptability to various gesture shapes and sizes.
- **Adaptive multi-scale attention module**: This module optimizes and adjusts the focus on different features through an attention mechanism. It allows the model to concentrate on the most critical features for the current gesture recognition task, improving accuracy and efficiency.

The integration of these modules enables the model to perform efficient and accurate gesture recognition, with the final prediction output through global pooling and fully connected layers. This structure optimizes the feature extraction process and allows the model to adapt to various complex application scenarios.

### Basic feature extraction module

**Fig 2:**
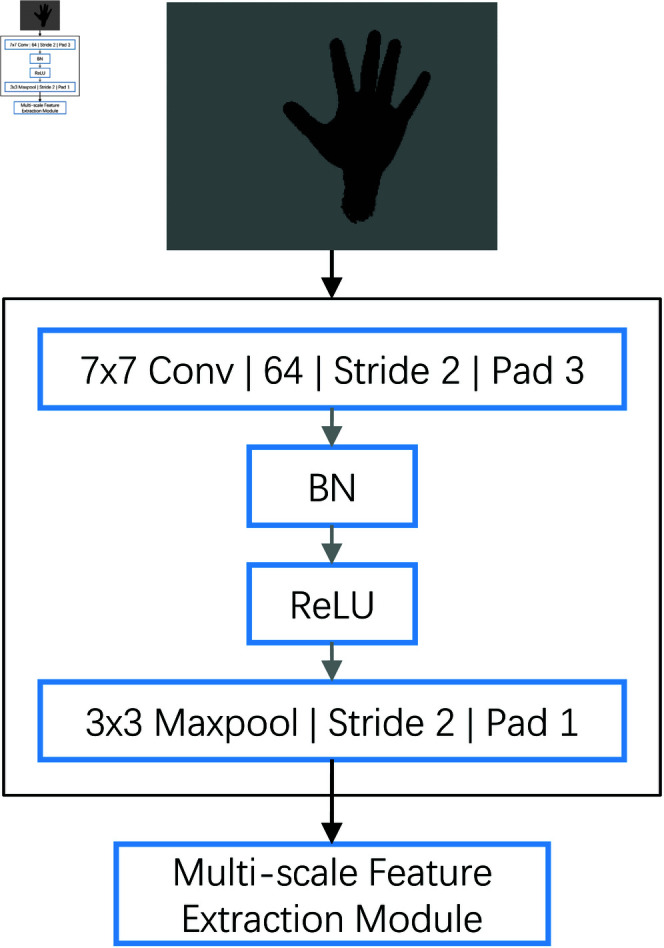
Architecture of the basic feature extraction module.

The Basic Feature Extraction Module plays a crucial role in our gesture recognition model. It extracts fundamental visual information from the input 128×128 pixel depth map, initially processing it through a 7×7 convolutional kernel to capture broad spatial information. By setting a stride of 2 and padding of 3, it optimizes the size and quality of feature mappings. Subsequently, the output features undergo batch normalization to improve training efficiency and stability, and ReLU activation enhances the non-linear characteristics and expressive power of the model, as illustrated in Fig 2.

In Fig 2, **BN** refers to Batch Normalization, a technique used to normalize the output of the previous layer by adjusting and scaling activations. This process helps reduce internal covariate shift, accelerates training, and improves the overall performance of the model.

To further compress the features and extract stronger spatial characteristics, we employ 3 × 3 MaxPooling. This step reduces the spatial dimensions of the feature maps, helping the model reduce computational load and increasing the abstraction level of the features. After these processing steps, the module outputs 64-channel 32 × 32 sized feature maps, providing a rich information foundation for subsequent deeper feature analysis and classification tasks. This design ensures the Basic Feature Extraction Module effectively captures key visual features from the original image, laying a solid foundation for recognizing complex gestures.

Specifically, the Basic Feature Extraction Module includes the following steps:

- **Convolution layer**: Uses a 7×7 convolutional kernel, stride of 2, and padding of 3 to extract initial visual features.
- **Batch normalization**: Standardizes the convolutional output to improve training efficiency and stability.
- **Activation function**: Employs ReLU to enhance non-linear characteristics.
- **MaxPooling layer**: Utilizes a 3 × 3 pooling kernel to reduce spatial dimensions and extract stronger spatial features.

### Multi-scale feature extraction module

**Fig 3:**
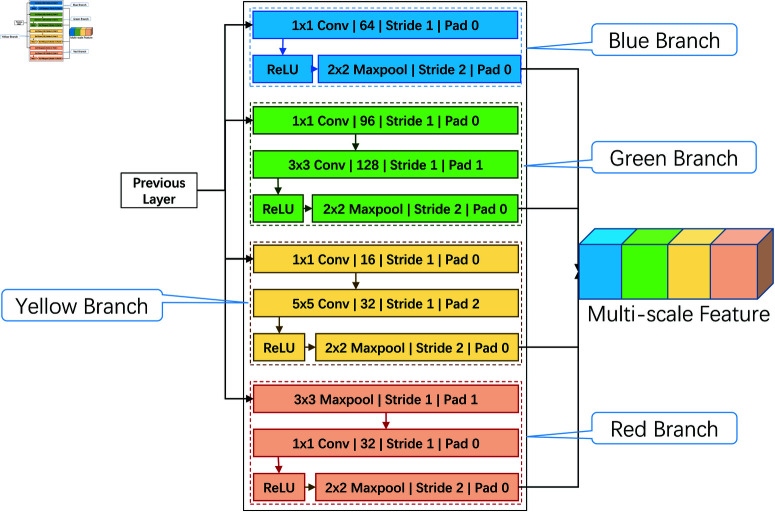
Architecture of the multi-scale feature extraction module.

To effectively capture multi-level visual information from local details to global structures, the Multi-Scale Feature Extraction Module is designed with inspiration from the Inception architecture. This module consists of four parallel branches, each focusing on different aspects of feature extraction through specific convolutional operations, as illustrated in Fig 3. The following subsections provide a detailed breakdown of each branch, including specific algorithms, techniques, and their functional roles.

#### Detailed branch operations

- **Blue branch**: This branch employs a 1 × 1 convolutional kernel with 64 filters to reduce the channel dimensions of the input features. This dimensionality reduction not only minimizes the computational load but also preserves essential spatial information. By applying a stride of 1 and no padding, the feature map size remains unchanged. The key advantage of using 1 × 1 convolutions here is the ability to compress feature dimensions, facilitating the easier processing of multi-scale features by subsequent layers. This operation is crucial for balancing computational efficiency and detailed feature extraction, laying the groundwork for effective multi-scale analysis.
- **Green branch**: This branch commences with a 1 × 1 convolution that reduces the feature dimensions to 96 channels, followed by a 3 × 3 convolution to capture fine-grained spatial features, such as edges and textures. The 3 × 3 convolution is applied with a stride of 1 and padding of 1, preserving the original spatial dimensions of the feature map. This combination allows the model to detect local patterns while maintaining computational efficiency. The sequential use of different convolutional kernels enables better detection of gesture-specific features, allowing the model to distinguish subtle variations in hand shapes or finger positions.
- **Yellow branch**: This branch commences with a 1 × 1 convolution for initial feature compression, reducing the channel number to 16. It is followed by a 5 × 5 convolution, which is crucial for expanding the receptive field and capturing larger spatial patterns. The 5 × 5 convolution is applied with a stride of 1 and padding of 2, ensuring that the feature map size remains consistent. The larger kernel size enables this branch to capture global patterns, enhancing its effectiveness in recognizing complex gestures that require a broader context. This capability is essential for understanding the overall structure of gestures, especially when different parts of the hand move simultaneously.
- **Red branch**: The red branch utilizes a 3 × 3 max pooling operation to enhance the model’s robustness against gesture position shifts. The max pooling is followed by a 1 × 1 convolution that readjusts the pooled features, combining them into a unified representation with 32 channels. This branch is designed to make the model more resilient to minor variations in gesture positioning, boosting generalization across different input variations. Max pooling also aids in emphasizing dominant features, which is important for identifying key gesture components.

Each branch output undergoes batch normalization and ReLU activation to improve network stability and non-linear feature learning. The outputs from all branches are concatenated along the channel dimension using the torch.cat operation, facilitating comprehensive utilization of multi-scale information. This feature fusion strategy ensures that the model can integrate information at different scales effectively, boosting the recognition of complex gestures. Furthermore, the concatenated features undergo additional batch normalization and ReLU activation to maintain consistency and enhance the effectiveness of the overall network training.

#### Multi-scale feature fusion

The final stage of the Multi-Scale Feature Extraction Module involves concatenating feature maps from all branches along the channel dimension, creating a unified representation that encapsulates multi-level information. This fusion step is implemented using the torch.cat function in PyTorch, which allows for efficient integration of features with varying receptive fields. The fused feature maps are then processed through a 1 × 1 convolution to reduce the channel number to 128, followed by global average pooling. This step ensures that the multi-scale information is compacted into a more manageable form, making it ready for downstream attention mechanisms. Specifically, the structure and parameters of the Multi-Scale Feature Extraction Module are detailed in Table 1.

**Table 1:** Multi-scale feature extraction module parameters.

Branch	Operation	Number of kernels	Stride	Padding	Activation function
Blue Branch	1x1 Convolution	64	1	0	ReLU
Green Branch	1x1 Convolution	96	1	0	-
	3x3 Convolution	128	1	1	ReLU
Yellow Branch	1x1 Convolution	16	1	0	-
	5x5 Convolution	32	1	2	ReLU
Red Branch	3x3 MaxPooling	-	1	1	-
	1x1 Convolution	32	1	0	ReLU

### Adaptive multi-scale attention module

**Fig 4:**
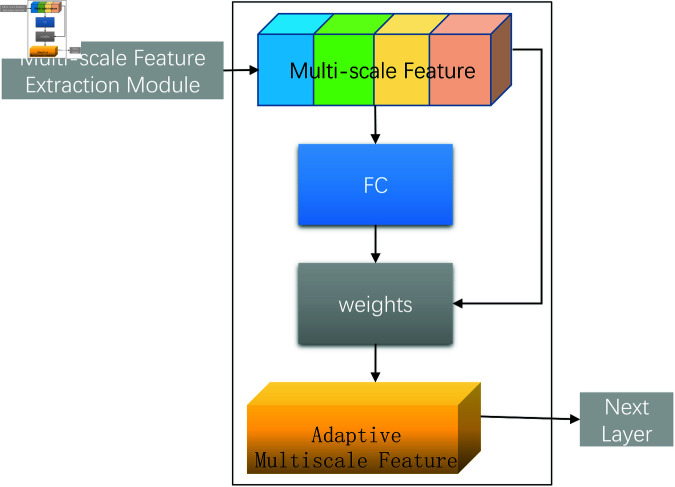
Architecture of the adaptive multi-scale attention module.

To enhance the efficiency and accuracy of feature extraction in the gesture recognition system, the Adaptive Multi-Scale Attention Module is introduced. This module leverages visual information at different scales to reinforce and optimize key feature recognition. It combines multi-scale feature fusion with a spatial attention mechanism to dynamically emphasize the most relevant features, as illustrated in Fig 4.

#### Feature fusion process.

In the feature fusion stage, adaptive multi-scale attention module employs the torch.cat operation to concatenate feature maps from different receptive fields along the channel dimension. This strategy allows the model to integrate multi-level information, forming a unified and rich feature representation that captures both fine local details and broader contextual information.

Let $F_{1},F_{2},\ldots ,F_{n}$ represent the feature maps obtained from different receptive fields, where *n* is the number of scales. The fused feature map $F_{\text{fused}}$ is computed as:

$$F_{\text{fused}}=\text{Concat}(F_{1},F_{2},\ldots ,F_{n}).$$ (1)

This fused feature map retains diverse spatial information, which is essential for accurate gesture recognition in varying conditions.

#### Spatial position weight calculation.

To calculate the spatial attention weights, adaptive multi-scale attention module uses a small fully connected network designed to evaluate the importance of each spatial position in the fused feature map. This attention mechanism highlights critical areas without altering the spatial dimensions of the feature map.

1. **Pooling Operations**: Adaptive multi-scale attention module first applies global average pooling and global max pooling to the fused feature map $F_{\text{fused}}$ along the channel dimension, generating two separate spatial descriptors:

$$F_{\text{avg}}(h,w)=\frac{1}{C}\sum_{c=1}^{C}F_{\text{fused}}(c,h,w),$$ (2)

$$F_{\text{max}}(h,w)=\max_{c=1}^{C}F_{\text{fused}}(c,h,w),$$ (3)

where *C* is the number of channels, and h,w denote the height and width of the feature map. This process extracts average and maximum responses across channels, representing global and local importance, respectively.

2. **Concatenation and Convolution**: The outputs $F_{\text{avg}}$ and $F_{\text{max}}$ are concatenated along the channel dimension:

$$F_{\text{concat}}=\text{Concat}(F_{\text{avg}},F_{\text{max}}).$$ (4)

This combined descriptor is then passed through a convolutional layer with a 7 × 7 kernel to generate the spatial attention map *A*_*s*_:

$$A_{s}=\sigma ({\text{Conv}}_{7\times 7}(F_{\text{concat}})),$$ (5)

where σ is the sigmoid activation function, which normalizes the output values to the range [0, 1]. The attention map *A*_*s*_ effectively assigns weights to different spatial positions, emphasizing more critical regions for gesture recognition.

#### Feature weight optimization.

In the feature weight optimization stage, the fused feature map $F_{\text{fused}}$ is refined by element-wise multiplication with the spatial attention map *A*_*s*_, resulting in an optimized feature map $F_{\text{optimized}}$:

$$F_{\text{optimized}}=F_{\text{fused}}⊙A_{s},$$ (6)

where ⊙ denotes element-wise multiplication. This operation adjusts the feature values based on their calculated importance, amplifying the more relevant features while suppressing less critical ones.

This dynamic weighting strategy allows adaptive multi-scale attention module to focus more effectively on key information, enhancing both the model’s sensitivity and its ability to recognize complex gestures. The spatial attention mechanism also ensures that the model adapts to different spatial distributions, improving recognition accuracy and robustness across various conditions.

In summary, the Adaptive Multi-Scale Attention Module combines multi-scale feature fusion with a robust spatial attention mechanism to optimize the final feature representation. This module significantly improves the efficiency and accuracy of gesture recognition, making it suitable for complex visual tasks.

## Experiments

### Dataset introduction

In the field of gesture recognition, the ICVL, NYU, and MSRA datasets are widely utilized benchmarks that offer unique challenges and features. These datasets allow researchers to assess and compare the performance of gesture recognition algorithms under different conditions.

- **ICVL dataset:** Introduced by the University of Birmingham in 2014, the ICVL dataset includes 330,000 training images and 1,596 testing images capturing various hand movements across ten different subjects. Images are captured using Intel’s Creative Interactive Gesture Camera, ensuring high-quality and consistency. Each image is annotated with precise locations of 16 joints in the hand, facilitating the training and validation of complex algorithms in controlled environments [28].
  The ICVL dataset covers a diverse range of hand gestures, making it particularly suitable for applications like sign language recognition, human-computer interaction, and robotic hand gesture understanding. Prior to model training, images are resized to 128 × 128 pixels, normalized, and augmented with random rotations and scaling to increase robustness. Joint positions are also normalized to facilitate model convergence during training.
- **NYU Hand Pose Dataset:** Released by New York University in 2014, this dataset includes 72,757 training samples and 8,252 testing samples captured from different viewpoints using a Kinect camera. It covers multiple subjects and provides annotations for 36 joint positions, although only 14 are commonly used for consistency in evaluation [29].
  The multi-view setup of the NYU dataset simulates real-world scenarios where gestures are observed from various angles, making it valuable for applications in augmented reality, immersive gaming, and gesture-based user interfaces. Images are first aligned using the wrist joint as a reference point, then cropped and resized to 128 × 128 pixels. Data augmentation techniques, such as rotation, scaling, and mirroring, are applied to enhance model generalization.
- **MSRA dataset:** Published by Microsoft Research Asia in 2015, it contains 76,500 images of hand gestures from nine different subjects, annotated with 21 joint positions. This dataset is particularly challenging due to its diversity and the precision required in joint prediction [30].
  The MSRA dataset’s diversity and variety of hand poses make it suitable for training models intended for gesture-based authentication systems, virtual reality environments, and human-robot interaction. The dataset undergoes standard preprocessing, including normalization and data augmentation. Each image is resized to 128 × 128 pixels, with joints normalized to fit within the image space. Random noise is added during training to simulate real-world sensor noise and enhance model robustness [30].

### Data preprocessing methods

Several key preprocessing methods were employed to enhance the robustness and performance of the gesture recognition models:

- **Data augmentation:** Incorporates various transformations such as random rotations, scaling, translations, and flipping to help the model generalize across different gesture appearances, orientations, and scales. This process improves model robustness by simulating diverse real-world conditions.
- **Batch Processing:** Implements torch.utils.data.DataLoader to optimize memory usage, computational efficiency, and parallel data loading, thus improving the model’s training speed.
- **Normalization:** Image data typically undergoes normalization, such as scaling pixel values to the [0, 1] range, or standardizing based on the dataset’s pixel mean and standard deviation, ensuring consistent input for the model and accelerating convergence during training.

### Evaluation metric

To evaluate the performance of the proposed gesture recognition model, **Mean 3D Error** is used as the primary evaluation metric. Mean 3D Error quantifies the average Euclidean distance between the predicted 3D joint positions and the ground truth positions across all joints in each frame. It is widely adopted in 3D hand pose estimation tasks due to its straightforward interpretation and relevance. This metric provides a direct reflection of the model’s accuracy in capturing the geometric relationships of hand joints, making it particularly suited for assessing localization precision. Lower Mean 3D Error values indicate a closer match between predicted and true joint positions, thereby demonstrating the model’s effectiveness in precise joint localization and supporting claims of improved accuracy in hand gesture recognition.

### Experimental setup

In the experimental setup, the models are trained using all available training samples from the datasets to ensure comprehensive learning and capture the full range of variations present in the data. The models are then evaluated on independent test sets, which have not been used during training, to assess their generalization capabilities. This approach allows for the evaluation of the model’s performance on unseen data and its ability to adapt to diverse gesture recognition tasks. Additionally, performance metrics such as accuracy, Mean 3D Error, and other relevant measures are computed to provide a comprehensive assessment of the model’s effectiveness.

### Ablation study

All models were trained and evaluated under identical data preprocessing, augmentation, and training setups to ensure consistency and minimize the impact of external variables. The preprocessing steps included normalization and random transformations, such as rotations and scaling, to simulate varying viewing conditions. Additionally, we employed data augmentation techniques such as flipping, cropping, and color jittering to further enhance the diversity of training data. This ensures the model’s robustness in real-world applications where viewing angles and lighting conditions can vary significantly.

#### Impact of multi-scale feature extraction on gesture recognition.

To evaluate the effectiveness of the Multi-Scale Feature Extraction Module, we compared the performance of our model, which integrates this module with the Adaptive Multi-Scale Attention Mechanism, against a baseline model. The baseline model uses standard 3 × 3 convolutional layers without multi-scale or attention mechanisms, providing a simplified architecture that serves as a benchmark for measuring the impact of our proposed enhancements.

Comparative experiments were conducted on the ICVL, NYU, and MSRA datasets. As shown in Table 2, the results indicate significant improvements in gesture joint prediction accuracy when the Multi-Scale Feature Extraction Module is integrated. The multi-scale approach allows our model to capture gestures with varying sizes and complexities, which traditional single-scale models often fail to handle effectively. This enhancement makes the model more adaptable to diverse real-world gesture variations.

**Table 2:** Performance comparison on ICVL, NYU, and MSRA datasets (Error in mm).

Method	ICVL	NYU	MSRA
Baseline	10.4	15.5	12.2
MultiScal	6.3	9.6	7.3

The significance of multi-scale extraction in gesture recognition lies in its ability to capture features at different levels of granularity, which is crucial for recognizing gestures that vary in size, complexity, and perspective. In gesture recognition, hands can appear at different scales depending on their proximity to the camera or their orientation, and multi-scale extraction allows the model to process these variations effectively. By analyzing the input at multiple scales, the model can detect both small, detailed features in gestures and larger, more abstract patterns, thereby improving overall recognition accuracy. Without multi-scale extraction, the model may miss essential information in gestures of varying sizes or struggle to generalize across diverse gestures, leading to reduced performance.

The effect of removing multi-scale feature extraction on various datasets was tested using the ICVL, NYU, and MSRA datasets. On the ICVL dataset, which features a variety of hand gestures at different scales, the model without multi-scale extraction showed a significant increase in error rates, from 6.3mm to 10.4mm, indicating its difficulty in handling gestures at various scales. Similarly, on the NYU dataset, the error rate increased from 9.6 mm to 15.5 mm, particularly when dealing with larger gestures. These results demonstrate that the multi-scale approach is essential for accurately capturing diverse gesture sizes and variations.

#### Effectiveness of the adaptive attention mechanism.

In this subsection, we focus on evaluating the effectiveness of the Adaptive Attention Mechanism in gesture recognition. To assess its impact, we conducted experiments comparing models with and without the attention mechanism. These experiments were carried out on the same datasets (ICVL, NYU, and MSRA) to ensure consistency and scientific validity of the results.

Both models, with and without the attention mechanism, utilized the same underlying network architecture to ensure a fair assessment of the attention module’s impact. The attention-equipped model integrates a spatial attention mechanism immediately after key convolutional layers, aiming to enhance the model’s capability to recognize critical gesture features. This spatial attention mechanism focuses on relevant regions of the input feature map, dynamically adjusting the focus based on the learning from previous layers.

The significance of the attention mechanism lies in its ability to help the model concentrate on the most important areas of the gesture, effectively improving recognition accuracy. In complex gesture recognition tasks, certain regions of the hand, such as the fingertips or palm, are more important than others, and the attention mechanism allows the model to dynamically adjust its focus to these regions. Without this attention mechanism, the model might distribute its resources evenly across all parts of the input, potentially ignoring crucial features and leading to lower recognition accuracy.

We also explored different variants of the attention mechanism, including both spatial and channel-wise attention, to identify the configuration that yields the best performance for gesture recognition tasks. The spatial attention mechanism emerged as the most effective, particularly in scenarios where gesture details are subtle or complex. This suggests that spatially aware attention plays a crucial role in identifying key gesture features.

Removing the attention mechanism has the following effect on various datasets. When the attention mechanism is removed, performance decreases across all three datasets (ICVL, NYU, and MSRA), as shown in Table 3. On the ICVL dataset, which includes a variety of hand gestures, the error rate increased from 6.3 mm (with attention) to 6.7 mm (without attention). Similarly, on the MSRA dataset, where gestures tend to have subtle details, the error rate rose from 7.3 mm to 9.2 mm without the attention mechanism. These results indicate that the attention mechanism significantly contributes to identifying the most relevant features of a gesture, especially when those features are complex or not easily distinguishable. In contrast, the results confirm that the model with the attention mechanism significantly reduces the error rates across all datasets compared to the baseline model. These improvements highlight the practical value of the attention mechanism in enhancing the accuracy and robustness of gesture recognition systems, especially in dynamically changing environments.

**Table 3:** Effectiveness of the attention module on gesture recognition accuracy compared between models with and without the attention mechanism on ICVL, NYU, and MSRA datasets (Error in mm).

Method	ICVL	NYU	MSRA
MultiScal	6.7	10.2	9.2
MultiScal + Attention (proposed)	6.3	9.6	7.3

The effectiveness in complex gesture scenarios is particularly evident when the Adaptive Attention Mechanism is applied to situations where gestures have complex or subtle details that are not easily detected by traditional convolutional operations. For example, in the MSRA dataset, gestures involving fine hand movements are significantly improved by attention, as the mechanism can focus on key features like the fingertips, which are often crucial for accurate gesture recognition. Without attention, the model struggles with these fine-grained details, leading to higher error rates.

**Fig 5:**
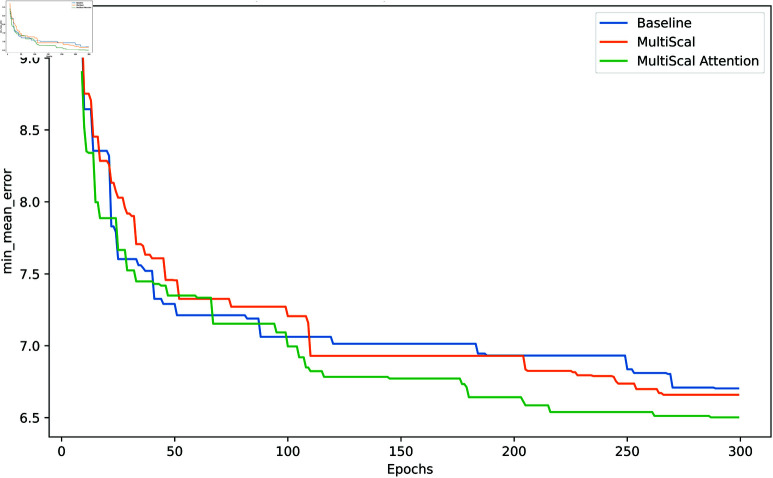
Comparison of minimum mean error across epochs for Baseline, MultiScal, and MultiScal attention methods on the MSRA dataset

Fig 5 illustrates the comparison of the minimum mean error across epochs for the Baseline, MultiScal, and MultiScal Attention methods on the MSRA dataset. From the figure, it is evident that the MultiScal and MultiScal Attention methods outperform the Baseline method in terms of error reduction over the course of training. Specifically, the MultiScal method demonstrates a consistent decrease in the minimum mean error throughout the epochs, showing its effectiveness in capturing multi-scale features. Furthermore, the MultiScal Attention method exhibits even greater improvement, particularly in the later stages of training, likely due to the spatial attention mechanism that helps the model focus on the most relevant parts of the input data, thereby enhancing recognition accuracy. This indicates that the combination of multi-scale feature extraction and attention mechanisms contributes significantly to improving model performance, particularly in complex tasks like hand gesture recognition.

The integration of both the Multi-Scale Feature Extraction Module and the Adaptive Attention Mechanism significantly enhances gesture recognition performance. The multi-scale approach helps capture gestures of various sizes and complexities, while the attention mechanism prioritizes critical areas of the input, leading to more accurate recognition results. Future research could explore combining these modules with temporal modeling techniques to further improve performance in dynamic scenarios.

### Comparison with state-of-the-art methods

The results in Table 4 showcase the performance of the proposed gesture recognition model in comparison to multiple state-of-the-art methods across the ICVL, NYU, and MSRA datasets. On the ICVL dataset, the model achieves an average 3D error of 6.3 mm, outperforming all other methods and underscoring its capacity to capture subtle hand gestures with high precision. This superior accuracy can be largely attributed to the combination of multi-scale feature extraction and adaptive attention, which together enable the model to balance fine local details with an understanding of broader hand structures. Such multi-scale processing is crucial in handling variations in hand shapes and gestures, which characterize ICVL’s diverse hand poses. The model’s performance demonstrates its capability to generalize well even in challenging scenarios, such as gestures with intricate poses and subtle movements.

**Table 4:** State-of-the-art Comparison of Hand Pose Estimation Methods on ICVL, NYU, and MSRA Datasets. We report the average 3D error in mm.

Method	ICVL	NYU	MSRA
LRF [31]	12.6	–	–
DeepModel [32]	17.0	11.6	–
Guo_Baseline [20]	8.4	14.6	–
DeepPrior++ [33]	8.1	12.3	9.5
REN [34]	7.5	13.4	–
DenseReg [35]	7.2	10.2	7.2
SHPR-Net [36]	7.2	9.4	7.8
HandPointNet [37]	6.9	10.5	8.5
Pose-REN [38]	6.8	11.8	8.6
CrossInfoNet [39]	6.7	10.0	7.8
Hand-Transforme [40]	6.5	9.8	7.6
A2J [41]	6.5	8.6	–
A2J-Transformer [42]	–	8.4	–
IPNet [43]	–	**7.2**	–
**Proposed**	**6.3**	9.6	**7.2**

A more detailed discussion on how the method outperforms others in practical, real-world applications. The proposed model’s superior performance extends beyond just numerical accuracy, showcasing its potential in practical, real-world applications. For example, the model’s ability to handle complex hand gestures and subtle movements, as demonstrated on the ICVL dataset, makes it highly suitable for real-time gesture recognition in interactive environments. Additionally, the multi-scale approach and attention mechanism allow the model to adapt to various lighting conditions, occlusions, and different hand poses, which are commonly encountered in real-world settings. This provides the model with an edge over other methods, which may struggle with such variations.

The experimental results on the NYU dataset show that the proposed model achieves a 3D error of 9.6 mm, indicating strong competitiveness in a dataset with complex characteristics such as occlusions and dynamic hand gestures. Although the error is slightly higher than IPNet’s 7.2 mm, it still demonstrates the model’s broad adaptability in handling diverse gestures. The increased error gap on the NYU dataset may be attributed to the presence of highly complex gestures or occlusions, where IPNet might have been specifically optimized to handle these challenging scenarios. Nonetheless, the proposed model remains competitive across a range of gesture types, confirming its robust performance.

On the MSRA dataset, the model achieves an average 3D error of 7.2 mm, matching the performance of DenseReg, one of the leading methods for this dataset. This performance reflects the proposed model’s adaptability and robustness across datasets with varying hand configurations and gesture types. The diverse nature of the MSRA dataset, which includes multiple subjects and a wide range of gesture poses, highlights the model’s ability to generalize across different conditions. The success on MSRA reaffirms the effectiveness of integrating multi-scale features and adaptive attention, as these components allow the model to process complex gestures while retaining high accuracy.

Although the model performs well across datasets, there are some limitations. In particular, the model may struggle with extreme occlusions or unfamiliar hand poses, which could affect its adaptability in unpredictable, real-world settings. Additionally, the computational demands associated with multi-scale and attention mechanisms might present challenges in resource-limited environments. Addressing these challenges in future work could involve optimizing the model for lower-quality data and exploring more lightweight, efficient architectures, making the model more applicable to real-time and embedded applications. Future optimizations could also focus on enhancing the model’s robustness to occlusions and rare hand poses, which would further broaden its real-world applicability.

Overall, the results demonstrate that the proposed model achieves high accuracy in diverse hand pose estimation tasks, confirming its effectiveness and robustness. The model’s consistent performance across different datasets underscores its potential in advancing gesture recognition technologies, providing a reliable basis for further innovations and applications in real-world settings.

### Conclusion

In this study, we developed an advanced gesture recognition model that integrates multi-scale feature extraction and attention mechanisms. The model was rigorously tested across three major gesture recognition datasets: ICVL, NYU, and MSRA, where it demonstrated superior performance compared to existing state-of-the-art technologies. The experimental results confirmed that multi-scale feature extraction effectively captures critical gesture features, enabling the model to address the diverse scale and complexity of hand gestures, while the attention mechanism enhances the model’s focus on significant information, helping to ignore irrelevant background noise. This dual approach allowed our model to excel in environments with complex backgrounds and dynamic changes, showcasing significant improvements in gesture recognition accuracy.

Future studies could explore integrating additional sensory data, such as RGB and infrared inputs, to increase robustness in low-light or occluded environments, where traditional vision-based systems may struggle. Potential applications of this model include human-computer interaction, virtual reality, sign language interpretation, and gesture-based control in robotics. Additionally, optimizing the model for deployment on edge devices or mobile platforms could enable broader use in real-time applications. Further research into extending the model to recognize more complex, multi-hand gestures or adapting it for other body parts, such as facial expression recognition, represents another promising direction. This would enhance the versatility of the model, making it applicable to a wider range of interactive systems and applications.
